# Current insights into the functional roles of ferroptosis in musculoskeletal diseases and therapeutic implications

**DOI:** 10.3389/fcell.2023.1112751

**Published:** 2023-02-03

**Authors:** Fan Zhang, Yuanliang Yan, Yuan Cai, Qiuju Liang, Yuanhong Liu, Bi Peng, Zhijie Xu, Wei Liu

**Affiliations:** ^1^ Department of Gynecology, Xiangya Hospital, Central South University, Changsha, China; ^2^ Department of Pharmacy, Xiangya Hospital, Central South University, Changsha, China; ^3^ National Clinical Research Center for Geriatric Disorders, Xiangya Hospital, Central South University, Changsha, China; ^4^ Department of Pathology, Xiangya Hospital, Central South University, Changsha, China; ^5^ Department of Orthopedic Surgery, The Second Hospital University of South China, Hengyang, China

**Keywords:** clinical implications, ferroptosis, musculoskeletal diseases, oxidative stress, therapeutic strategies

## Abstract

Ferroptosis is a novel type of cell death associated with iron accumulation and excessive lipid peroxidation. Elucidating the underlying molecular mechanisms of ferroptosis is intensively related to the development and treatment of multiple diseases, including musculoskeletal disorders. Moreover, *in vitro* and *in vivo* studies have shown the importance of oxidative stress in musculoskeletal conditions such as osteoporosis, osteoarthritis, rheumatoid arthritis, and osteosarcoma. Ferroptosis-derived clinical management of musculoskeletal diseases offers tremendous and attractive opportunities. Notably, ferroptosis agonists have been proven to enhance the sensitivity of osteosarcoma cells to conventional therapeutic strategies. In this review, we have mainly focused on the implications of ferroptosis regulation in the pathophysiology and therapeutic response of musculoskeletal disorders. Understanding roles of ferroptosis for controlling musculoskeletal diseases might provide directions for ferroptosis-driven therapies, which could be promising for the development of novel therapeutic strategies.

## 1 Introduction

Ferroptosis is a regulated cell death induced by iron-correlated lipid peroxidation. The crucial impetus of ferroptosis is the phospholipids peroxidation and formation of cellular membranes (Hadian and Stockwell, 2020). Ferroptosis induction through oxidative perturbations of the intracellular microenvironment controlled by glutathione (GSH) peroxidase 4 (GPX4) has been reported, essentially triggered by Fe^2+^ accumulation and lipid peroxidation (Su et al., 2020).

Numerous studies have demonstrated the importance of ferroptosis in human diseases, such as nervous system disorders, cardiovascular diseases, and cancers (Wei et al., 2020). Additionally, emerging evidence has supported the notion that ferroptosis and musculoskeletal diseases are closely linked (Zhang et al., 2022a); however, the underlying mechanisms require further investigation. Accordingly, ferroptosis and other types of programmed cell death have been studied extensively for their involvement in musculoskeletal diseases (Hwang and Kim, 2015; Spel and Martinon, 2020; Zhao et al., 2021a).

**FIGURE 1:**
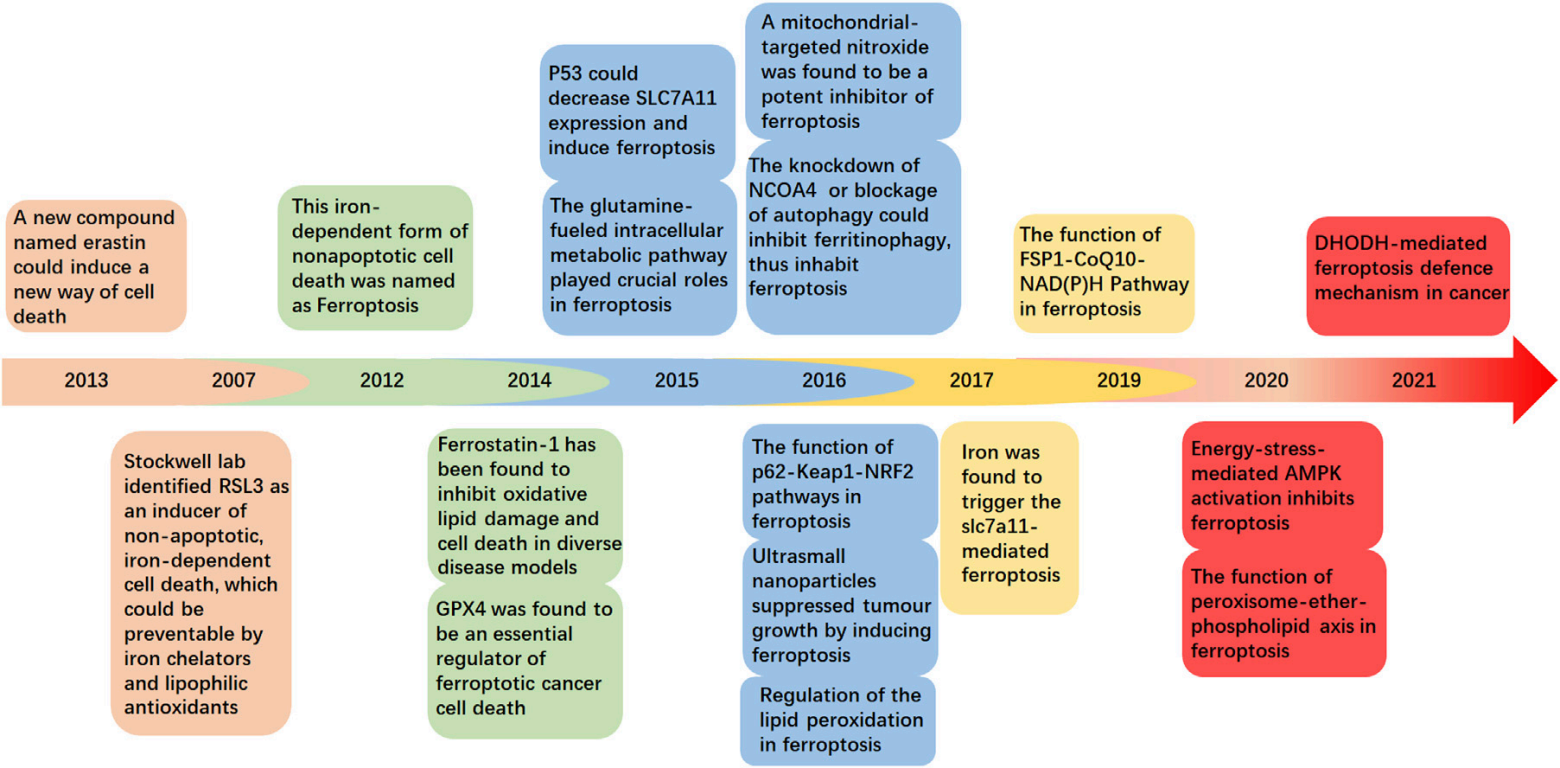
Timeline showing ferroptosis-related key discoveries.

In this review, we have summarized and discussed the profiles and clinical implications of ferroptosis related to musculoskeletal disorders, which are currently under intensive investigation (Figure 1). These discoveries could provide a more detailed understanding of musculoskeletal diseases to determine potential therapeutic strategies.

## 2 Regulatory mechanisms of ferroptosis

**FIGURE 2:**
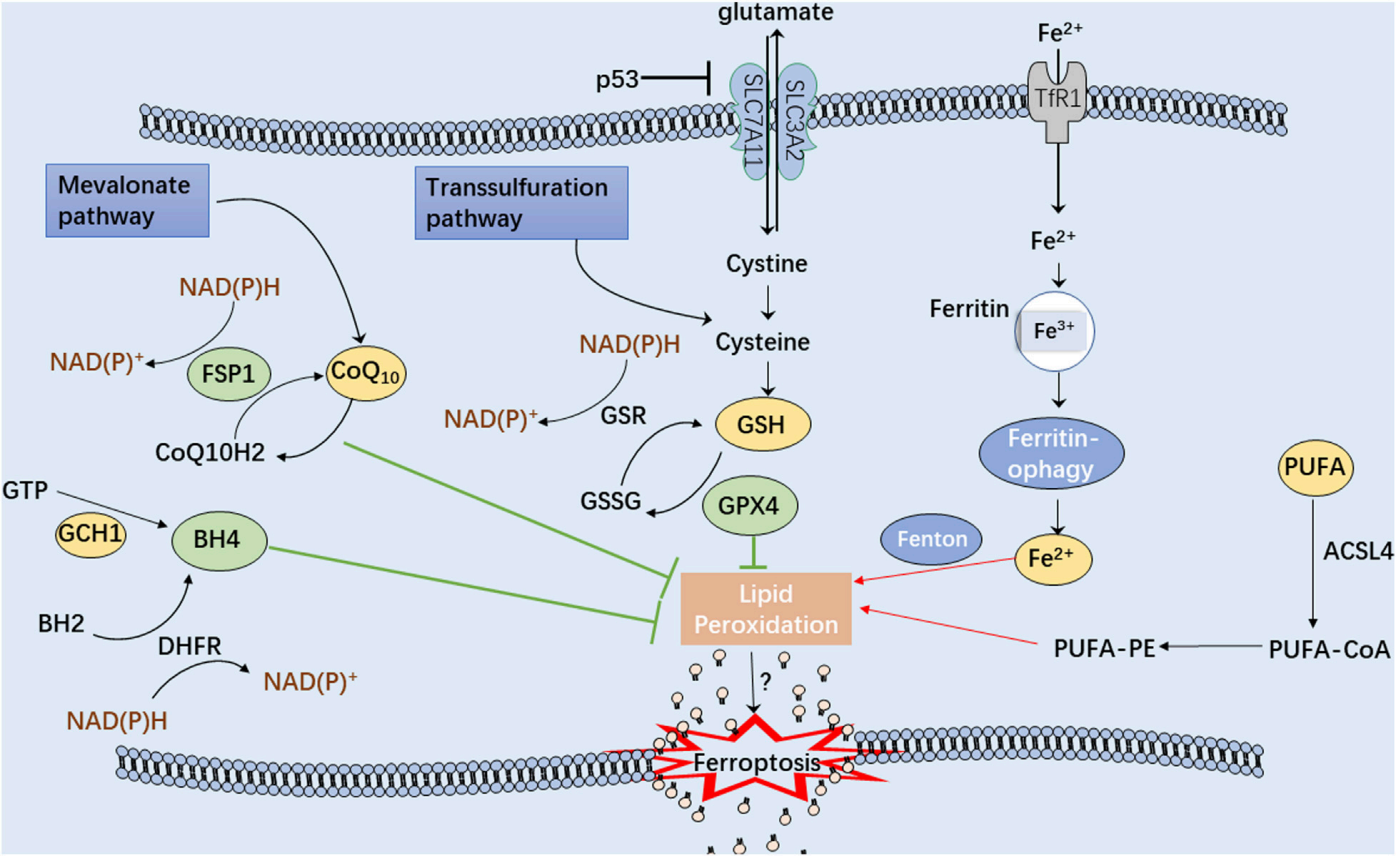
A schematic illustration showing the main mechanisms of the ferroptosis process. The green arrows represent three antioxidant systems: Xc-GSH-GPX4 pathway, FSP1-CoQ10-NAD(P)H pathway, and GCH1-BH4 pathway. Iron metabolism disturbance and polyunsaturated fatty acids (PUFA) peroxidation, marked with red arrows, significantly contribute to cellular ferroptosis.

Ferroptosis can be distinguished from other programmed cell death modes having characteristics of mitochondrial damage, such as mitochondrial swelling, decreased cristae, and an increase in autophagosomes (Friedmann Angeli et al., 2014). Because of the uncontrollable production of lipid peroxides, polyunsaturated fatty acid (PUFA) hydroperoxides accumulate within the phospholipid membranes, providing the fundamental lipid component necessary for ferroptotic cell death that leads to enhanced membrane permeability and cell swelling (Yang et al., 2016; Agmon et al., 2018). Additionally, in ferroptotic cells, the antioxidant defense systems, Xc-, ferroptosis suppressor protein 1 (FSP1), GTP cyclohydrolase 1 (GCH1), and other pathways, are critically responsible for the regulation of lipid peroxidation and ferroptosis (Figure 2). Intriguingly, ferroptosis-associated cell death may spread to neighboring cells in waves, further triggering their death (Riegman et al., 2020). Therefore, safeguarding these cells and rebuilding the cell membrane lipids is crucial before undergoing cellular ferroptosis (Doll et al., 2017).

### 2.1 Iron metabolism

Iron metabolism homeostasis may influence cell sensitivity to ferroptosis (Yu et al., 2020; Zheng and Conrad, 2020) because iron could produce large amounts of reactive oxygen species (ROS) through the Fenton reaction, thus, exacerbating oxidative damage to cells (Yoshida et al., 2019). Therefore, controlling iron transport and storage is critical to regulating ferroptosis (Chen et al., 2020). Hypoxia-inducible factor (HIF), generated under hypoxic conditions, has been reported to regulate the functioning of iron-transferring proteins, including transferrin receptor (TfR), divalent metal transporter 1 (DMT1) and iron transport protein (FPN) (Tacchini et al., 1999; Taylor et al., 2011; Xu et al., 2017; Yang et al., 2018). Moreover, ferritinophagy mediated by nuclear receptor coactivator 4 (NCOA4) has been implicated in maintaining iron homeostasis through converting ferritin into intracellular iron (Santana-Codina and Mancias, 2018; Fuhrmann et al., 2020; Santana-Codina et al., 2021). Iron chelators, including deferoxamine (DFO) and ciclopirox, have been demonstrated to prevent ferroptosis by forbidding the production of oxidized lipid species (Stockwell et al., 2017). Recent studies have suggested the functional roles of aberrant iron overload or iron deficiency on the osteoblast ferroptosis, which might contribute to the occurrence of osteoporosis (Toxqui and Vaquero, 2015; Jiang et al., 2022; Xu et al., 2022). It has been observed that intracellular iron overload caused by ferrous ammonium citrate (FAC) could improve ferroptosis sensitivity by downregulating the expression of Wnt target genes, Lef1, Bmp4, Smad6, and Cyclin D1, consequently blocking the development of mesenchymal stem cells into osteoblasts (Luo et al., 2022). Moreover, iron overload reduces the activity of end-plate chondrocytes and knee chondrocytes, hence participating in intervertebral disc degeneration and osteoarthritis (OA) (Cai et al., 2021; Chen et al., 2022a). Wang’s group constructed a mouse model of iron overload and found that iron overload could induce cartilage calcification in a dose-dependent manner (Wang et al., 2022a). Previous research has demonstrated that DMT1 plays a pivotal role in the prevention of iron overload and ferroptosis in chondrocytes in OA (Jing et al., 2020). In the OA mouse model induced by ferroptosis activator erastin, the iron overload in chondrocytes could facilitate the development of arthritis by downregulating type II collagen and upregulating matrix metalloproteinase 13^33^. Besides, distinct from normal cells, osteosarcoma cells display an exuberant iron demand due to their malignant proliferative characteristic and high cellular iron concentration (Xue et al., 2021). The salazosulfapyridine-induced ferroptosis in K7M2 osteosarcoma cells could be significantly reversed after depletion of iron (Liu et al., 2022a). Therefore, targeting iron metabolism could be served as an appealing strategy for regulating the cell death of osteosarcoma.

### 2.2 Lipid peroxidation

Uncontrolled lipid peroxides production due to oxidative stress generally causes mitochondrial lipid peroxidation and damage, resulting in ferroptosis. Based on the differences in hydrocarbon chain saturation, fatty acids can be divided into three categories: saturated fatty acids (SFAs), monounsaturated fatty acids (MUFAs), and PUFAs (Tang et al., 2021a). The density of PUFAs significantly impacts the degree of lipid peroxidation, thereby determining the sensitivity of ferroptosis (Stockwell et al., 2017). The free PUFAs, including arachidonic acid (AA) and adrenic acid (ADA), are catalyzed by acyl-CoA synthetase long-chain family member 4 (ACSL4) to produce AA/AdA-CoA derivatives. Next, AA/AdA-CoA and membrane PE are catalyzed by lysophosphatidylcholine acyltransferase 3 (LPCAT3) to obtain AA/AdA-PE (Doll et al., 2017; Kagan et al., 2017; Zou et al., 2019; Kagan et al., 2020). Upon the overactivation of lipid peroxide synthesis, PUFA depletion can alter the establishment and operation of lipid membranes (Hassannia et al., 2019). Thus, ACSL4 and LPCAT3 have been identified as crucial targets for ferroptosis regulation due to their involvement in PUFA catalysis. A recent study revealed that the natural flavonoid glycoside, baicalin, could suppress the expression of ACSL4 to prevent cell ferroptosis (Fan et al., 2021). Similarly, rosiglitazone, an ACSL4 inhibitor, has been reported to prevent acute kidney injury induced by ferroptosis (Wang et al., 2022b).

### 2.3 Xc-GSH-GPX4 pathway

In mammals, the cyst(e)ine-GSH-GPX4 signaling axis has been identified as the primary regulatory system for ferroptosis. ROS are the byproducts of aerobic metabolism, and unrestrained ROS production significantly contributes to cellular ferroptosis (Sun et al., 2018). GSH is an essential antioxidant tripeptide consisting of glutamic acid, cysteine, and glycine (Kuang et al., 2020). The system Xc− and transsulfuration pathways are the two main pathways that produce cysteine for GSH synthesis (Liu et al., 2020; Martis et al., 2020). System Xc− has the function of transporting cystine into cells and constitutes the subunit solute carrier family 7 member 11 (SLC7A11) and solute carrier family 3 member 2 (SLC3A2) (Lewerenz et al., 2013). After cellular entry, cystine is oxidized to cysteine, which is utilized in GSH synthesis (Ursini and Maiorino, 2020). Besides, the transsulfuration pathway is related to the reciprocal conversion of cysteine to homocysteine (McBean, 2012). GPX4 (a selenium-containing protein) plays a crucial role in the inhibitory function of GSH on cellular ferroptosis by reducing the phospholipid hydroperoxide production (AA/AdA-PE-OOH) to the corresponding phosphatidyl alcohol (PLOH) (Ingold et al., 2018; Zhang et al., 2021). Another molecule, erastin (a classical inducer of ferroptosis), can inhibit extracellular cystine from entering cells by blocking the system Xc−, thus, lowering intracellular GSH expression and inducing ferroptosis (Zhao et al., 2020). Further research has revealed more compounds that participate in the regulation of the system Xc−pathway, such as RSL3, FIN56 and FINO2 (Yang et al., 2014; Gaschler et al., 2018; Wen et al., 2019; Yu et al., 2021).

### 2.4 FSP1-CoQ10-nad(P)H pathway

Ferroptosis and phospholipid peroxidation are also controlled by FSP1/ubiquinone (CoQ10)/NAD(P)H pathway, which is a GPX4-independent non-mitochondrial coenzyme Q10 (CoQ10)-based antioxidant system. FSP1 can catalyze the production of ubiquinol, the reduced form of CoQ10, at the cell membrane *via* NAD(P)H and subsequently traps lipid peroxides to inhibit ferroptosis (Doll et al., 2019). Interestingly, another study reported that CoQ10 could also suppress ferroptosis *via* the endosomal sorting complex required for transport (ESCRT)-III membrane repair, which is a distinct mechanism independent of ubiquinol (Dai et al., 2020). In addition, UBIA prenyltransferase domain-containing protein 1 (UBIAD1) is an essential antioxidant enzyme involved in the biosynthesis of non-mitochondrial CoQ10. UBIAD1/CoQ10 inhibition has been proposed as a potential strategy for treating melanoma by promoting lipid peroxidation and ferroptotic cell death (Arslanbaeva et al., 2022).

### 2.5 GCH1-BH4 pathway

GCH1/tetrahydrobiopterin (BH4)/phospholipid signaling axis has been recently reported to function as a highly potent ferroptosis suppressor. Moreover, the GCH1-BH4 pathway has been demonstrated as an endogenous antioxidant pathway independent of GSH-GPX4 (Kraft et al., 2020). BH4 serves as a cofactor in redox metabolism and regulates the metabolism of monoamines and aromatic compounds (Vancassel et al., 2022; Vasquez-Vivar et al., 2022). Aberrantly expressed BH4 is capable of trapping oxidative free radicals and preventing the production of lipid peroxides. Furthermore, dihydrofolate reductase (DHFR) can efficiently regenerate BH4 to defend against lipid peroxidation and ferroptosis (Soula et al., 2020). In addition, Hu et al. have delineated that genetic or pharmacological inhibition of GCH1 could improve the sensitivity of colorectal cancer cells to ferroptosis-inducing agents by downregulating BH4 metabolism (Hu et al., 2022a).

### 2.6 Other pathways regulating ferroptosis

Mounting evidence indicates the existence of other anti-lipid oxidation routes participating in the regulation of ferroptosis. For instance, an *in vivo* study suggests that iPLA2 inhibition may increase the susceptibility of cancer cells to p53-driven ferroptosis, which is independent of GPX4 (Chen et al., 2021a). In another report, Zou et al. identified P450 oxidoreductase (POR) as a significant enzyme contributing to lipid peroxidation and ferroptosis in cancer cells in response to distinct ferroptotic stress, using CRISPR-Cas9-mediated suppressor screens (Zou et al., 2020). Furthermore, energy stress led to the inactivation of AMP-activated protein kinase (AMPK) signaling that impaired the protective effects of energy stress on ferroptosis, contributing to ferroptosis-associated diseases (Lee et al., 2020). These findings collectively imply that clarifying the underlying molecular signals for ferroptotic cell death could provide a viable possibility to develop ferroptosis-based strategies, thus, impacting the therapeutic efficacy in human diseases.

## 3 Modulators that induce or inhibit ferroptosis in musculoskeletal diseases

**FIGURE 3:**
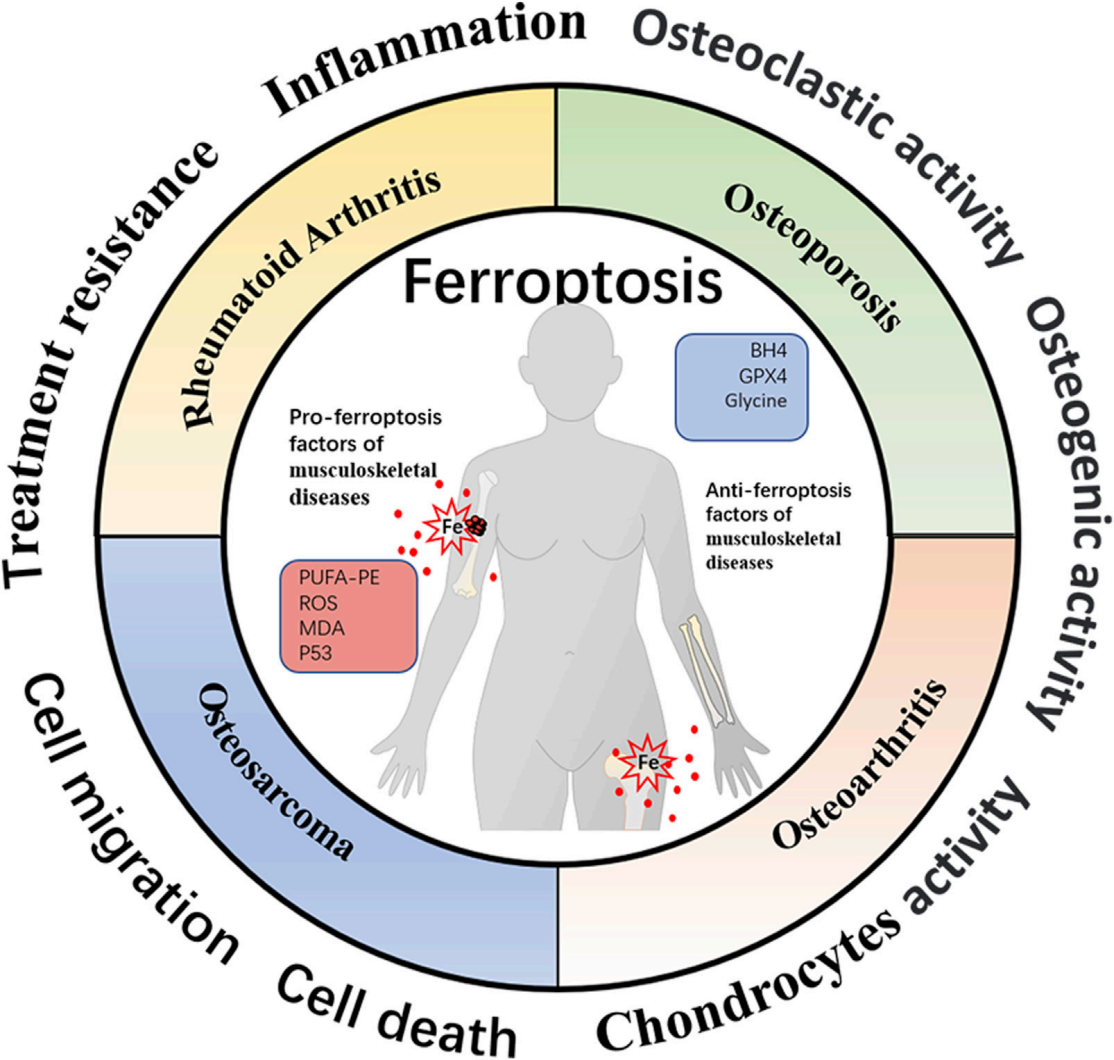
Schematic representation of ferroptosis-associated biomarkers in four different musculoskeletal diseases. The profiles of ferroptosis-related molecules have been proven to be well‐established mediators affecting musculoskeletal disease progression and therapeutic response. The organic hallmarks present the biological functions of musculoskeletal diseases, including inflammation, cell death, cell migration, treatment resistance, etc.

Recent emerging reports have demonstrated the critical role of cellular ferroptosis in musculoskeletal diseases (Figure 3). Additionally, several ferroptosis regulators have shown promising therapeutic effects in the experimental models of human musculoskeletal disorders (Table 1). During oxidative stress conditions, the ferroportin (FPN)-dependent iron homeostasis weakened ferroptosis in bone cells *in vitro* and *in vivo*, suggesting the protective role of FPN in the pathogenesis of skeletal diseases (Lu et al., 2021).

**TABLE 1:** List of genes in different tissue and their respective function.

Gene	Function	Tissue type	References
HIF-2α	potentiates cell ferroptosis *via* lipid oxidation, ROS accumulation and ferroptosis regulators	chondrocytes	Zhou et al. (2021)
MTF1	regulates intercellular iron overload and prevention of ferroptosis in cells	chondrocytes	Lu et al. (2021)
HO-1	overexpresses HO-1 may activate HGHF-induced osteocyte ferroptosis	osteocyte	Yang et al. (2022a)
P53	decreases the expression of P53 could reverse the bavachin-induced osteosarcoma ferroptosis	osteosarcoma	Luo et al. (2021)
ZFP36	overexpresses in osteosarcoma patients, could be a potential predictive marker for osteosarcoma	osteosarcoma	Song et al. (2021)

### 3.1 Osteoarthritis

Being an important process in inflammatory diseases (Keuters et al., 2021; Chang et al., 2022), ferroptosis-based strategies have presented a long-time challenge for the clinical development of OA. In a rat OA model, treatment with interleukin (IL)-1β resulted in the decrease of collagen II and GPX4 and an increase in matrix metalloproteinases (MMPs), ROS and ion concentration, contributing to extracellular matrix (ECM) degradation and ferroptosis sensitivity (Lv et al., 2022). Additionally, two ferroptosis inhibitors, ferrostatin-1 (a lipophilic antioxidant) and DFO (an iron chelator), have demonstrated protective effects on OA cells, further supporting the predominant role of ferroptosis in OA pathology (Miao et al., 2022). Under inflammatory and iron overload conditions, intra-articular injection of ferrostatin-1 enhanced ferroptosis resistance in chondrocytes, leading to OA progression (Yao et al., 2021). In another report, treatment with IL-1β and erastin induced ferroptosis in mouse chondrocytes marked with excessive accumulation of lipid ROS and malondialdehyde (MDA), which could be reversed by DFO (Guo et al., 2022).

Recently, aberrant activation of HIFs displayed a functional role in controlling cellular metabolism and was involved in the progression of inflammatory diseases (Gonzalez et al., 2018). Under inflammatory conditions, D-mannose impaired glucose metabolism by inhibiting the succinate-induced activation of HIF-1α and lipopolysaccharide (LPS)-mediated activation of macrophages (Torretta et al., 2020). Other studies reported that HIF-2α (encoded by Epas1) influenced the metabolic phenotypes of cartilage and OA pathogenesis (Saito et al., 2010; Yang et al., 2010). The nicotinamide phosphoribosyl transferase (NAMPT) has been reported to be a direct target for HIF-2α; its overexpression could significantly increase the expression levels of matrix-degrading enzymes, causing cartilage destruction and accelerated OA development (Yang et al., 2015). Zhou et al. documented that mannitol reduced the sensitivity of chondrocytes to ferroptosis by inhibiting HIF-2α, thereby protecting chondrocytes and alleviating OA progession (Zhou et al., 2021). However, the detailed role of ferroptosis in OA pathogenesis and the therapeutic response has not been deeply explored. Therefore, ferroptosis-mediated occurrence and development of OA require more clinical and experimental research.

Identifying potential therapeutic agents for OA treatment and elucidating their underlying molecular mechanisms would be beneficial for developing novel strategies against OA. A recent study has shown that icariin (a major pharmacological component of a natural compound isolated from *Epimedium*) suppressed the expression of OA-related risk factors, such as IL-1β, MMPs, and GRP78, and improved the treatment efficacy against OA (Pan et al., 2017). Moreover, *in vitro* experiments have demonstrated that icariin could activate the Xc-/GPX4 signaling pathway to inhibit ferroptosis in response to the LPS-stimulated inflammation, thereby protecting the synovial cells from death (Luo and Zhang, 2021).

### 3.2 Osteoporosis

Osteoporosis is a major public health concern known to cause serious health risks and economic costs. Several factors have been demonstrated to contribute to the development of osteoporosis, such as inflammatory response, metabolic disorders, and genetic conditions (Ebeling et al., 2022). A few recent studies have established the involvement of ferroptosis in the progression of osteoporosis and the disruption of the balance between osteoclastic and osteogenic activities (Liu et al., 2022b; Gao et al., 2022; Yan et al., 2022). Another report showed that dexamethasone could weaken the activity of intracellular antioxidant systems through downregulation of the SLC7A11/GPX4 signaling, thereby enhancing the ferroptosis sensitivity of MC3T3-E1 and MOLY4 preosteoblasts and contributing to glucocorticoid-induced osteonecrosis (Sun et al., 2022). In a mouse model of steroid-induced osteoporosis, Lu et al. observed that the extracellular vesicles from bone marrow-derived endothelial progenitor cells (EPC-EVs) could alleviate the pathological changes related to osteoporosis by suppressing a series of ferroptosis associated features of osteoblasts (Lu et al., 2019). In a similar report, endothelial cell-secreted exosomes (EC-Exos) suppressed dexamethasone-induced osteoblast ferroptosis and reversed the inhibitory effect of osteoblast activity (Yang et al., 2021). Altogether these findings confirm the crucial role of ferroptosis in regulating the osteogenic activity during osteoporosis. Moreover, ferroptosis suppression could be a promising therapeutic strategy for osteoporosis management.

Ferroptosis induction by a high-fat and high-glucose diet may be the initiating factor for diabetic osteoporosis. High-fat and high-glucose conditions have resulted in osteoblastic ferroptosis by promoting METTL3-dependent m6A methylation of apoptosis signal-regulating kinase 1 (ASK1) and activating p38 signaling. On the contrary, METTL3 knockdown significantly abrogated the activation of ASK1-p38 signaling axis, resulting in ferroptosis attenuation and diabetic bone loss (Lin et al., 2022). In addition, mitochondrial ferritin (FtMt) is a key protein responsible for maintaining iron homeostasis and protecting cells from iron imbalance-induced death (Wang et al., 2021a). Increased FtMt expression can reduce cellular ROS concentration and inhibit ferroptosis in type 2 diabetic osteoporosis. Conversely, decreased FtMt expression may cause mitochondrial autophagy, further disturbing iron homeostasis and activating cell ferroptosis (Wang et al., 2022c). Furthermore, Yang et al. identified high levels of several pro-ferroptotic genes, such as heme oxygenase-1 (HO-1), in murine models of diabetic osteoporosis. Targeting HO-1 prohibited the occurrence of lipid peroxidation in osteocytes and effectively ameliorated the deterioration of bone trabeculae (Yang et al., 2022a). Moreover, the activation of Nrf2/HO-1 signaling pathway aided in the antagonistic effect of ferroptosis inhibitor melatonin against the high glucose-induced osteoporosis (Ma et al., 2020). These results suggest that pro-ferroptotic genes could serve as potential biomarkers for clinical therapy of diabetic osteoporosis.

### 3.3 Rheumatoid arthritis

Rheumatoid arthritis (RA) is a systemic inflammatory disease whose pathogenesis is often characterized by the infiltration of pro-inflammatory synovial fibroblasts, elevated frequency of synovial osteoclasts, and progressive joint destruction (Smolen et al., 2016; Zhao et al., 2022). Multiple strategies to inhibit the proliferation of synovial fibroblasts and reinstate synovial homeostasis in RA could provide promising therapeutic directions (Chen et al., 2022b; Ji et al., 2022; Sandhu and Thelma, 2022). Recent studies have reported that the increased risk of RA disease might be associated with the dysfunction of the antioxidant system in fibroblast-like synoviocytes (FLS). Additionally, the molecular characteristics of ferroptosis play an important role in maintaining the death and survival balance of synovium in RA patients, suggesting the involvement of ferroptosis in the pathogenesis and development of RA (Luczaj et al., 2016; England et al., 2018). Using a collagen-induced arthritis mouse model, Wu et al. reported that the combination of imidazole ketone erastin (a ferroptosis inducer) and etanercept (a tumor necrosis factor (TNF) inhibitor) could induce ferroptotic cell death and inhibit cell proliferation in synovial fibroblasts, thereby attenuating the RA development (Wu et al., 2022). In another report, glycine administration significantly induced the S-adenosyl methionine-dependent promoter methylation of GPX4 and decreased the expression of ferritin heavy chain 1 (FTH1) in FLS cells, followed by activation of FLS ferroptosis hindering RA development (Ling et al., 2022). These findings indicate the potential of ferroptosis-inducing compounds for exploitation as therapeutic candidates for RA patients.

Ferroptosis incentive influences RA progression; thus, explicating its underlying molecular mechanism could capture significant attention for RA treatment. Aberrant expression of FSP1, a molecular biomarker for ferroptosis inhibition (Bersuker et al., 2019), may be associated with RA development. Mechanistically, FSP1 overexpression interferes with the underlying signaling for lipid ROS generation, including TNF-α and p38/JNK signaling pathway. Based on the inhibition of ROS-induced peroxidation, FSP1 could protect chondrocytes from ferroptosis and delay the process of RA (Xie et al., 2021). In addition, serotransferrin (TF, a ferroptosis-inducer) (Hong et al., 2021) has been implicated in stimulating the anti-RA response. A corresponding study suggested the reduction of serum TF levels in antirheumatic drug-resistance patients (Chen et al., 2021b).

## 4 Approaches specifically targeting ferroptosis in musculoskeletal tumors

### 4.1 Ferroptosis inhibits osteosarcoma progression

Exploring the ferroptosis-related molecular mechanisms could provide new directions to study carcinogenesis and treatment response (Lin et al., 2021a; Qu et al., 2021). Previous studies have demonstrated the significance of targeting ferroptosis in anti-osteosarcoma treatment (Zhao et al., 2021b) (Table 2). Another intriguing research pointed out that iron zinc finger protein 36 (ZFP36), a ferroptosis-related gene, was significantly overexpressed in osteosarcoma patients, displaying a negative correlation with progression-free survival and overall survival; targeting ZFP36 could serve as a promising therapeutic biomarker for osteosarcoma patients (Song et al., 2021). Besides, traditional medicines, such as bavachin, might act as prospective drugs for the treatment of osteosarcoma patients (Luo et al., 2021). For example, administration of curcumin analogue EF24 in osteosarcoma U2OS and Saos-2 cells induced ferroptotic cell death characterized by increased concentration of intracellular MDA, lipid ROS, and ferric ions. However, the knockdown of ferroptosis-associated HMOX1 inversely attenuated the EF24-induced cytotoxicity effects (Lin et al., 2021b). Treatment with theaflavin-3,3′-digallate (TF3) could raise ROS levels and activate MAPK signaling pathways, causing ferroptotic cell death in OS cells MG63 and HOS (He et al., 2022). In addition, under hypoxia conditions, the prodrug tirapazamine was reported to decrease proliferation and induce ferroptosis in osteosarcoma 143B and U2OS cells; the biological functions were linked to the inhibition of SLC7A11 and GPX4 (Shi et al., 2021). Wang et al. (2022d) constructed the MH-PLGA-IR780 nanoparticles with homologous targeting ability, and found that MH-PLGA-IR780 could improve the photodynamic therapy (PDT)-induced ferroptosis sensitivity in human OS cell HOS, thus effectively killing tumors. Knockdown of Yes1 associated transcriptional regulator (YAP) in combination with ferroptosis induction obviously enhanced the sensitivity to pyropheophorbide-methyl ester-mediated PDT (MPPa-PDT) in HOS cells (Zhan et al., 2022).

**TABLE 2:** Interventions and reagents targeting ferroptosis for osteosarcoma.

Reagents/targets	Osteosarcoma cell lines	Mechanisms	Biological effects on ferroptosis	Refs
Bavachin	MG63, HOS	Inhibiting STAT3 and increasing P53 to inhibit SLC7A11	Inducing ferroptosis	Luo et al. (2021)
EF24	U2os, Saos-2	Upregulation of HMOX1 expression and inhibition of GPX4 expression	Inducing ferroptosis	Lin et al. (2021b)
TF3	MG63, HOS	Disturbing the redox balance, and activating the ROS-related MAPK signaling pathway	Inducing ferroptosis and apoptosis	He et al. (2022)
Tirapazamine	HOS, 143B, U2os	Inhibiting SLC7A11 and GPX4	Inducing ferroptosis	Shi et al. (2021)
MH-PLGA-IR780 NPs nanoplatform	HOS	Promoting ROS accumulateion *via* photodynamic therapy	Inducing ferroptosis and apoptosis	Wang et al. (2022d)
miR-1287-5p	U2os, Saos-2	Inhibiting GPX4	Inducing ferroptosis and increasing sensitivity to cisplatin	Xu et al. (2021)
KDM4A	143B, HOS	Inducing H3K9me3 demethylation to increase SLC7A11 transcription	Inhibiting ferroptosis and increasing sensitivity to cisplatin	Chen et al. (2021c)
Ursolic acid	143B, HOS	Inducing NCOA4- mediated ferritin autophagy and intracellular ferrous ions overload	Inducing ferroptosis and increasing sensitivity to cisplatin	Tang et al. (2021b)
Combination of erastin, RSL3 and STAT3	MG63, Saos-2	Disturbing STAT3/Nrf2/GPx4 signaling pathway	Inducing ferroptosis and increasing sensitivity to cisplatin	Liu and Wang (2019)
Nano-carrier loaded with ferrate and doxorubicin	Saos-2	Inducing reoxygenation and glutathione-depletion	Inducing ferroptosis and apoptosis	Fu et al. (2021)

### 4.2 Ferroptosis reduces chemotherapy resistance in osteosarcoma

The efficacy of osteosarcoma therapy is often limited due to the development of resistance. Ferroptosis modulation using pharmacological agents or genetic methods is required to sensitize osteosarcoma cells to enhance treatment benefits. MiR-1287-5p inhibited the activity of GPX4 *via* binding to its 3′-untranslated region, hence inducing ferroptosis in osteosarcoma cells. Notably, the intervention of miR-1287-5p led to osteosarcoma cells being more sensitive to cisplatin chemotherapy (Xu et al., 2021). In addition, KDM4A, a histone demethylase with the function of demethylating H3K9me3 in the promoter region of SLC7A11, inhibited ferroptosis-related cell death in osteosarcoma. Moreover, decreased KDM4A expression resulted in increased cell ferroptosis, attenuated migration ability, and enhanced cisplatin sensitivity in osteosarcoma 143B and HOS cells (Chen et al., 2021c). In another study, Tang et al. showed that ursolic acid, a natural component derived from radix *Actinidiae chinensis*, promoted ferroptosis sensitivity of osteosarcoma 143B and HOS cells by decomposing ferritin and caused intracellular iron accumulation through the activation of ferritinophagy. Additionally, ursolic acid effectively enhanced the cytotoxic effects of cisplatin on osteosarcoma cells (Tang et al., 2021b). Likewise, the combination of ferroptosis agonists (erastin and RSL3) and STAT3 (signal transducer and activator of transcription 3) inhibitors synergistically impaired the cisplatin resistance in osteosarcoma MG63 and Saos-2 cells (Liu and Wang, 2019). Recently, nanomedicine-based therapeutic methods have been applied to overcome treatment resistance and efficiently inhibit tumor development by inducing ferroptosis of tumor cells (Wang et al., 2021b). Fu et al. constructed a nano-carrier loaded with ferrate and doxorubicin that could initiate the Fenton reaction to promote ROS overproduction and iron-dependent ferroptotic cell death, thus overcoming chemotherapy resistance in osteosarcoma cells both *in vitro* and *in vivo* (Fu et al., 2021).

### 4.3 The ferroptosis-based strategies in other musculoskeletal tumors

Growing knowledge of ferroptosis in musculoskeletal tumors has led to the discovery of potential molecular targets for the development and identification of anti-cancer drugs. Moreover, the ferroptosis displays a crucial regulatory effect on the pathogenesis and therapeutic response of other musculoskeletal tumors, such as rhabdomyosarcoma and fibrosarcoma (Liu et al., 2022c).

Of note, Efimova et al. (2020) revealed that, in fibrosarcoma MCA205 cells, ferroptosis induced by RSL3 could cause HMGB1 and ATP-dependent immunogenic cell death (ICD). According to other studies, indocyanine green (ICG)-based PDT combined with NIR could cause ferroptosis in HT1080 fibrosarcoma cells (Tseng et al., 2022). Interestingly, the photosens (PS)-based PDT on MCA205 fibrosarcoma cells could also induce cellular ferroptosis and ICD (Turubanova et al., 2019). In addition, the advancements of nanomedical technology bring new hopefulness for the clinical manegment of musculoskeletal tumors. Zhou and his colleagues prepared siRNA@ABMBP-COF containing HK2 inhibitor 3-bromopyruvate and SLC7A11 siRNA. They found that siRNA@ABMBP-COF could induce ferroptosis and apoptosis in HT1080 fibrosarcoma cells by downregulating SLC7A11 and HK2, suggesting the significant anti-tumor capacity in a tumor-bearing nude mouse model (Zhou et al., 2022). Iron oxide nanoparticles (IONP-GA/PAA) enrobed with gallic acid and polyacrylic acid may be effective in killing HT1080 fibrosarcoma cells *via* HMOX1-dependent ferroptosis (Fernandez-Acosta et al., 2022). The agents, DO264 and salazosulfapyridine, cause ferroptosis in HT-1080 fibrosarcoma cells by inhibiting ABHD12 and system Xc-, resectively (Kathman et al., 2020; Yin et al., 2022). Additionally, the inhibitors targeting mitochondrial NADP-dependent isocitrate dehydrogenase (IDH2) and lysosome could efficiently prevent ferroptosis in HT-1080 fibrosarcoma cells by increasing GSH levels and decreasing intracellular ROS generation (Torii et al., 2016; Kim et al., 2020). Erastin or RSL3 have been shown to cause ferroptosis in rhabdomyosarcoma cells, which could be blocked by ferroptosis inhibitor ferrostatin-1 (Schott et al., 2015). Meanwhile, PKC inhibitors could also prevent erastin-induced ferroptosis in rhabdomyosarcoma cells (Dachert et al., 2020). These studies might provide novel insights into the ferroptosis-based therapeutic strategies in musculoskeletal tumors.

## 5 Discussion and future remarks

Ferroptosis has been considered a complex and anomalous metabolic process associated with pathological alterations in a wide array of disorders. Although ferroptosis-related research in musculoskeletal conditions is still in the infancy stage, the strategies interfering with disease progression through modulation of the ferroptosis pathway display enormous clinical potential (Table 3), including enhanced viability of osteoblasts and diminished therapeutic resistance.

**TABLE 3:** Interventions and reagents targeting ferroptosis for musculoskeletal diseases.

Intervention methods or reagents	Mechanism	Effects on cells	References
ferrostatin-1	attenuating the cytotoxicity, ROS and lipid-ROS accumulation and ferroptosis related protein expression changes induced by IL-1β and FAC and facilitated the activation of Nrf2 antioxidant system to inhibit ferroptosis	chondrocytes	Yao et al. (2021), Miao et al. (2022)
DFO	abrogating ROS and lipid ROS accumulation and the increase in MDA, promoting nuclear factor E2-related factor 2 (Nrf2) antioxidant system activation to inhibit ferroptosis	chondrocytes	Yao et al. (2021), Guo et al. (2022)
D-mannose	inhibiting HIF-2α and production of macrophage IL-1β, reduce the sensitivity of cells to ferroptosis	chondrocytes	Zhou et al. (2021)
Icariin	activate the Xc-/GPX4 signaling pathway to inhibit ferroptosis	chondrocytes	Luo and Zhang (2021)
EPC-EVs	reversing dexamethasone treatment-induced alterations in cysteine and several oxidative injury markers to suppressing the ferroptosis pathway	osteoblasts	Lu et al. (2019)
EC-Exos	suppress dexamethasone-induced ferroptosis	osteoblasts	Yang et al. (2021)
Melatonin	inhabiting ferroptosis and osteoporosis through activating of Nrf2/HO-1 signaling pathway	osteoblasts	Ma et al. (2020)
IKE and TNF inhibitors	inducing fibroblasts ferroptosis to slow the collagen-induced arthritis	fibroblasts	Wu et al. (2022)
Glycine	increasing the expression level of SAM, induces GPX4 promoter methylation and decrease of FTH1 in FLS, activates RA FLS ferroptosis	fibroblast-like synoviocytes	Ling et al. (2022)
FSP1	interfering with the TNF-α/ROS-positive feedback loop on the basis of inhibition of ROS, while restraining chondrocytes ferroptosis and delaying the development of RA	chondrocytes	Xie et al. (2021)
MiR-1287-5p	inhibiting the activity of GPX4 *via* binding to its 3′-untranslated region, hence inducing the ferroptosis in osteosarcoma cells	osteosarcoma	Xu et al. (2021)
KDM4A	demethylating H3K9me3 in the promoter region of SLC7A11, inhibiting ferroptosis-related cell death in osteosarcoma	osteosarcoma	Chen et al. (2021c)
Ursolic Acid	activating osteosarcoma cell ferroptosis through decomposing ferritin and causing intracellular iron ion accumulation	osteosarcoma	Tang et al. (2021b)
a nanomedicine loaded with ferrate and doxorubicin	modulating the tumor microenvironment and deplete GPX4 to induce osteosarcoma cells ferroptosis	osteosarcoma	Fu et al. (2021)
EF24	causing overexpression of HMOX1 and then inhibit GPX4-related ferroptosis	osteosarcoma	Lin et al. (2021b)
Tirapazamine	inducing osteosarcoma cell ferroptosis *via* inhibiting SLC7A11	osteosarcoma	Shi et al. (2021)

Considering the chronic nature of a large proportion of musculoskeletal diseases, the drugs activated dose should be maintained for an extended period to treat the abnormal state. Therefore, local drug delivery with precise dose control should be monitored carefully to reduce adverse effects. For instance, the non-specific toxicity and rapid metabolism of DFO restrict its clinical application. *In vivo* experiments have shown that ROS-responsive polymeric nanogels containing DFO moieties (rNG-DFO) could reduce iron-mediated oxidative stress and enhance the safety profile of DFO (Liu et al., 2018). Abbina et al. demonstrated that iron-chelating nanoconjugate could be used to slow down drug metabolism rates and ameliorate the drug toxicity of DFO (Abbina et al., 2019). Thus, further investigation of ferroptosis-modulating drugs and the corresponding drug delivery methods is crucial in exploiting new treatment schedules for musculoskeletal disorders.

Ferroptosis has been defined as a distinguished type of programmed cell death compared to others, such as apoptosis, necroptosis, and pyroptosis; however, ferroptosis may not occur in isolation but is intricately linked with other forms of cell death in musculoskeletal conditions. For instance, NCOA4 induced ferritinophagy, a selective type of autophagy leading to iron accumulation, which could further induce osteoclast ferroptosis and reduce bone resorption in ovariectomy mouse (Ni et al., 2021). To adequately understand the effect of ferroptosis-associated diseases, the relationships among various cell deaths remain to be addressed. Verifying their roles in musculoskeletal disorders as mutually antagonistic or facilitative would provide new insights and prospects for disease treatment.

Emerging signaling pathways, such as Xc-GSH-GPX4, FSP1-CoQ10-NAD(P)H, and GCH1-BH4, have been confirmed to be crucial for ferroptosis regulation in musculoskeletal diseases. In the cellular model of TNF-α-induced RA, the bioactive peptide G1dP3 was demonstrated to be a potential therapeutic agent against RA; this effect was correlated with the increased p53-mediated ferroptosis in synovial fibroblasts (Hu et al., 2022b). However, the involvement of ferroptosis regulatory molecules, such as p53, in musculoskeletal diseases still requires further elaboration. Previous studies have indicated that CoQ10 has tremendous potential for application in treating musculoskeletal diseases, including RA (Abdollahzad et al., 2015; Jhun et al., 2020) and OA (Chang et al., 2020). In addition, Zhang et al. (2022b) reported that CoQ10 could improve oxidative stress, as indicated by the increased MDA concentration. Another report by Yang et al. supported the functional regulatory effects of CoQ10 in ferroptosis, controlling sodium iodate-induced pathologies *in vitro* and *in vivo* (Yang et al., 2022b). However, the efficacy of CoQ10 as a ferroptosis-modulating agent against musculoskeletal disorders is not yet fully elucidated. Determining whether targeting CoQ10 could regulate ferroptosis to treat musculoskeletal diseases or any modulators of CoQ10-related signaling pathways could be exploited as potential ferroptosis-modulating therapeutic agents indicate a promising role of ferroptosis in controlling cell death and may provide new avenues for identifying treatment targets for musculoskeletal diseases.

## Glossary

GSH: glutathione
GPX4: glutathione peroxidase 4 (GPX4)
PUFA: polyunsaturated fatty acid
FSP1: ferroptosis suppressor protein 1
GCH1: GTP cyclohydrolase 1
ROS: reactive oxygen species
HIF: hypoxia-inducible factor
TfR: transferrin receptor
DMT: divalent metal transporter 1
NCOA4: nuclear receptor coactivator 4
DFO: deferoxamine
SFAs: saturated fatty acids
MUFAs: monounsaturated fatty acids
AA: arachidonic acid
ADA: adrenic acid
ACSL4: acyl-CoA synthetase long-chain family member 4
LPCAT3: lysophosphatidylcholine acyltransferase 3
SLC7A1: solute carrier family 7 member 11
SLC3A2: solute carrier family 3 member 2
ESCRT: endosomal sorting complex required for transport
UBIAD1: UBIA prenyltransferase domain-containing protein 1
CoQ10: coenzyme Q10
DHFR: dihydrofolate reductase
POR: P450 oxidoreductase
AMPK: AMP-activated protein kinase
FPN: ferroportin
OA: osteoarthritis
MMPs: matrix metalloproteinases
ECM: extracellular matrix
MDA: malondialdehyde
LPS: lipopolysaccharide
NAMPT: nicotinamide phosphoribosyl transferase
ASK1: apoptosis signal-regulating kinase 1
FtMt: mitochondrial ferritin
HO-1: heme oxygenase-1
RA: rheumatoid arthritis
FLS: fibroblast-like synoviocytes
FTH1: ferritin heavy chain 1
TF: serotransferrin
ZFP36: zinc finger protein 36.
